# Preoperative CA19-9 and GGT ratio as a prognostic indicator in ampullary carcinoma

**DOI:** 10.1186/s12876-022-02623-0

**Published:** 2023-03-13

**Authors:** Rui-Qiu Chen, Zhi-Lei Zhang, Yu-Ming Jia, Rui-Xiang Chen, Li Peng

**Affiliations:** 1 Department of Hepatobiliary Surgery, The Fourth Affiliated Hospital of Hebei Medical University, Hebei, China; 2 Jianhua Campus, Hebei Medical University, shijiazhuang, China

**Keywords:** Carbohydrate antigen199, Gamma-glutamyltransferase, Ampullay carcinoma, Predictive value, Nomogram

## Abstract

**Background and aims:**

In recent years, more and more inflammatory indicators have been studied to predict the long-term survival of patients with ampullary carcinoma (AC) after radical resection, but these prognostic indicators are still controversial. Therefore, based on previous inflammation scores, this study established a novel, easily accessible, more feasible and more predictive prognostic marker [Carbohydrate antigen199 to gamma-glutamyltransferase ratio (CA19-9/GGT)] to better assess the prognostic significance in AC patients undergoing radical resection.

**Methods:**

Overall survival (OS) and recurrence-free survival (RFS) were analyzed by Cox regression model. Correlation between CA19-9/GGT and clinicopathological variables were analyzed by Chi-squared test, Fisher ' s exact test, independent sample t test and Mann–Whitney *U* test. The performance of prognostic indexes is compared by the consistency index (C-index). The prediction accuracy of nomogram is further confirmed by calibration curve and decision curve analysis (DCA).

**Results:**

CA19-9/GGT was an independent risk factor affecting OS [*P* = 0.001, hazard ratio (HR) 2.459, 95% confidence intervals (CI) 1.450–4.167] and RFS (*P* = 0.002, HR 2.333, 95% CI 1.371–3.971) in multivariate analysis. The optimal cut-off value of CA19-9/GGT was 0.14. In CA19-9/GGT correlation analysis, high risk group (> 0.14) was significantly associated with poor prognosis. The predictive performance of CA19-9/GGT (OS: C-index = 0.753, RFS: C-index = 0.745) was confirmed to be superior to other prognostic indicators according to the C-index. Compared with the simple AJCC staging system, the Nomogram prediction model (OS: C-index = 0.787, RFS: C-index = 0.795) established by the combination of CA19-9/GGT and AJCC 8th TNM staging system has higher prediction accuracy.

**Conclusions:**

CA19-9/GGT was an independent prognostic indicator after radical resection of AC. Incorporating CA19-9/GGT into the AJCC TNM staging system optimized the prediction accuracy of the TNM staging system, and further verified the predictive value of CA19-9/GGT.

## Introduction

Ampullary carcinoma (AC) is a rare malignant tumor in the gastrointestinal tract, accounting for about 0.2–0.5% of all gastrointestinal malignancies, and about 6–17% of periampullary tumors [1, 2]. Pancreaticoduodenectomy (PD) is currently the best treatment option for patients with ampulla cancer who have surgical indications [3, 4]. Although the prognosis of ampullary carcinoma after radical resection is better than that of other periampullary carcinomas, the prognosis of some patients is worse than expected [5].

Serum carbohydrate antigen (CA) 19-9 is a glycoprotein of cancer cells, which has been widely used as the preferred tumor marker for the initial diagnosis of ampullary carcinoma, and is also an important indicator for evaluating the recurrence and long-term prognosis of patients [6, 7]. However, serum CA19-9 is easily interfered by other factors. When there is biliary obstruction or coexisting diseases, the serum CA19-9 level will be abnormally increased, resulting in a decline in the reference value of preoperative serum CA19-9 for the prognosis and survival assessment of patients [8].

Serum γ-glutamyltransferase (GGT) is a trypsin widely distributed on the surface of glandular and ductal epithelial cells, mainly secreted by the liver, and it is also the most sensitive liver enzyme to reflect biliary obstruction and liver injury [9]. Studies have shown that GGT has a certain correlation with the prognosis and survival of various digestive tract malignancies, but due to a variety of other diseases and conditions (such as renal insufficiency, diabetes, pancreatitis, myocardial infarction, obesity and alcohol intake) Serum GGT levels are elevated and therefore lack specificity [9–11].

In the past decade, the inflammation-based scores have emerged as novel prognostic indexes for a variety of malignant tumors [12–20], such as neutrophil-to-lymphocyte ratio (NLR), platelet-to-lymphocyte ratio (PLR), and has been favored by more and more scholars. However, the predictive value of these inflammation-based scores remains controversial [12–14, 19]. In view of the above considerations, CA19-9/GGT ratio was used to evaluate whether it will become a simple, accessible, and more predictive prognostic indicator for AC patients after radical resection.

Therefore, the purpose of this study is to analyze whether CA19-9/GGT has a certain predictive value for the prognosis and survival of patients with radically resected AC. Meanwhile, compared with other predictive indexes, whether CA19-9/GGT is a potential prognostic indicator with higher predictive value. Furthermore, CA19-9/GGT predictors combined with the existing American Joint Committee on Cancer (AJCC) 8th Tumor-Node-Metastasis (TNM) staging to establish Nomogram prediction model, whether CA19-9/GGT can further confirm its own predictive value, refine the existing AJCC staging system and improve AJCC staging system prediction value, thus providing additional evidence for surgeons to make better clinical decisions before surgery, and theoretically help predict the prognosis of patients with survival.

## Materials and methods

### Study standards and design

This study retrospectively analyzed 162 patients with ampullary carcinoma who underwent radical pancreaticoduodenectomy in the Fourth Affiliated Hospital of Hebei Medical University from January 2011 to April 2021. Inclusion criteria: (1) Preoperative evaluation reached the indication of radical resection, and radical pancreaticoduodenectomy was performed; (2) Postoperative pathological diagnosis was ampullary carcinoma; exclusion criteria: (i) Patients died within one month after operation; (ii) Patients who died of non-ampullary cancer; (iii) the clinical and follow-up data of the patient were missing. According to electronic medical records, the general characteristics, perioperative characteristics, preoperative laboratory indexes, postoperative complications and pathological examination of patients were recorded, and many predictive parameters that may affect long-term survival were counted, such as CA19-9/GGT, PLR, NLR, Albumin to Alkaline Phosphatase Ratio (AAPR), Glucose to Lymphocyte Ratio (GLR), Gamma-glutamyltransferase to Platelet Ratio (GPR) and albumin to globulin ratio (AGR). At the same time, we use the AJCC 8th edition TNM staging system as a reference for clinical pathological staging [21].

### Patient follow-up

Patients were followed up regularly after surgery and Postoperative follow-up included clinical and laboratory examinations. Regular follow-up once a month within 6 months after operation; If there is no recurrence within 6 months, follow-up every 3 months; Patients without recurrence in 3 years were followed up every 6 months. The time interval between the operation date and the first recurrence date or the last follow-up date was defined as RFS. The OS interval is from the date of surgery to the date of death or the last follow-up. The end point of follow-up was May 2022. All patients or their families were informed consent.

### Statistical analysis

Continuous variables are expressed as mean ± standard deviation; The receiver operating characteristic (ROC) curve was used to determine the optimal cut-off value of routine blood indexes and prediction parameters; Chi-square test, Fisher's exact test and Mann–Whitney U test were used to compare the clinicopathological features of the two groups. In the comparison between groups of measurement data, independent sample t test is used for normal distribution, and rank sum test is used for non-normal distribution. χ^2^ test or Mann–Whitney *U* test was used for the comparison between groups of enumeration data. Kaplan–Meier method was used to calculate the overall survival curve and non-recurrence survival curve; comparison of OS and RFS between CA19-9/GGT subgroups by log-rank test; The association of relevant variables with OS and RFS was assessed using Cox regression model; *P* < 0.05 indicated that the difference was statistically significant; the predictive ability of prognostic indicators was evaluated and compared by C-index. Nomogram was drawn by combining AJCC 8th TNM stage with CA19-9/GGT in R. C-index, calibration curve and Decision Curve Analysis (DCA) were used to compare and evaluate Nomograms with the simple AJCC 8th edition TNM staging system. Statistical analysis were performed by the software statistical package for social sciences version 26.0 (SPSS, Chicago, IL), R project version 4.0.5 (http://www.r-project.org/) and Medcalc software (version 15.2.2.0; Ostend, Belgium). The optimal cut-off values for prognostic indicators and inflammation-based scores were determined using X-tile3.6.1 software (Y ale University, New Haven, CT, USA). Time-dependent ROC was depicted using KM method via the survival ROC package in R. The nomogram was computed with the rms package in R.

## Results

### Patient data analysis

During the study period, a total of 174 patients with AC underwent radical pancreatoduodenectomy, including 2 patients diagnosed with ampullary neuroendocrine tumors, 3 patients died of accidents, 1 patient died within 30 days after surgery, and 6 patients were excluded due to incomplete information or lost to follow-up, so a total of 162 patients with ampullary cancer were enrolled. Among 162 patients, 138 patients were found to have jaundice symptoms after the first examination after hospitalization. The chief complaints during interrogation included 67 cases of jaundice, 53 cases of upper abdominal discomfort, 11 cases of fever, 11 cases of loss of appetite, 8 cases of abnormal urine, 4 cases of nausea and anorexia, 5 cases of tumors found by examination, 1 case of waist and back discomfort, 1 case of skin pruritus, 1 case of emaciation. Among 138 jaundice patients, 38 patients received preoperative jaundice reduction treatment, including 12 patients received Endoscopic Retrograde Cholangiao-Pancreatography, Endoscopic sphincterotomy, Endoscopic Nasal Bile Drainage (ERCP + EST + ENBD) jaundice reduction treatment, and 26 patients received Percutaneous Transhepatic Cholangial Drainage (PTCD) jaundice reduction treatment. 87 patients (53.7%) received postoperative adjuvant therapy, but the specific therapeutic regimen was not known. The median follow-up time was 40 months [Inter-Quartile Range (IQR) = 21–58 months]. The 1-, 3-, 5-year OS and RFS were 87%, 60.5%, 44.1% and 67.9%, 54.3%, 40.6%, respectively. The specific clinicopathological features of the patients are detailed in Table 1.

**Table 1:** Clinicopathological characteristics of patients with AC: survival analysis and COX regression analysis

Variables	Patients (n=162)	OS			RFS		
		Univariate (*P* value)	Multivariate (*P* value)	Multivariate (HR 95%CI)	Univariate (*P* value)	Multivariate (*P* value)	Multivariate (HR 95%CI)
*General character*							
Gender, male/female	95/69	**0.023**	NS		**0.027**	NS	
Age, years (x±s)	61.9 ± 8.4	0.003			0.098		
Drinking history yes/no	59/105	0.592			0.664		
Hepatobiliary and Pancreatic Coexisting Diseases yes/no	53/109	0.879			0.721		
Underlying diseases yes/no	69/93	0.651			0.961		
Hypertension/Diabetes/Coronary heart disease/Cerebral infarction	43/30/9/13						
*Perioperative characteristics*							
Preoperative jaundice reduction yes/no	38/124	0.698			0.867		
Blood transfusion yes/no	139/23	0.911			0.832		
Preoperative/Intraoperative/Postoperative	16/29/129						
Complications yes/no Pancreatic-fistula/Gastrointestinal hemorrhage/	88/74	0.253			0.311		
	72/16/8/13						
Abdominal-infection/Gastric-emptying disorder							
Postoperative adjuvant therapy yes/no	87/75	0.069			0.091		
*Pathological features*							
Tumor size (cm)> 2.6 / ≤2.6	47/115	**0.004**	NS		**0.002**	NS	
Differentiation		** < 0.001**	**<0.001**		** < 0.001**	**<0.001**	
High differentiation	11		NS				
Medium / low differentiation	116/35		**0.013**	6.372 (1.470–27.610)		**0.004**	4.76 (1.077–21.030)
Lymph node invasion yes/no	52/110	**0.001**	NS		** < 0.001**	**0.022**	1.762 (1.089–2.849)
Perineural invasion yes/no	60/102	**0.001**	NS		**0.004**		
Vascular invasion yes/no	17/145	0.139			0.090		
AJCC 8th edition 0/ IA, B/IIA, B/IIIA, B	2/57/51/52	** < 0.001**			** < 0.001**		
*Conventional hematological indexes*							
TBIL (µmol/L) > 256.6 / ≤256.6	37/125	** < 0.001**			** < 0.001**	NS	
Albumin (g/L) > 43.55 / ≤43.55	16/146	0.403			0.38		
Alkaline phosphatase (U/L)> 430.45 / ≤430.45	74/88	0.343			0.305		
GGT (U/L)> 86.85 / ≤86.85	148/14	0.589			0.645		
CA19-9 (U/L)> 74.97 / ≤74.97	95/67	** < 0.001**	**<0.001**	3.401 (1.953–5.922)	** < 0.001**	**<0.001**	3.477 (1.991–6.073)
*Prognostic indicator*							
PLR>266.44 / ≤266.44	36/126	**0.027**	NS		0.055		
NLR>3.51 / ≤3.51	67/95	0.128			0.157		
AAPR>0.28 / ≤0.28	27/135	0.608			0.627		
GPR>4.06 / ≤4.06	37/125	**0.01**	NS		**0.011**	NS	
GLR>507.2 / ≤507.2	73/89	**0.005**	NS		**0.003**	NS	
AGR>0.035 / ≤0.035	126/36	**0.003**	**<0.001**	6.474 (2.972–13.850)	**0.005**	**<0.001**	5.58 (2.576–12.093)
CA19-9/GGT>0.14 / ≤ 0.14	93/69	** < 0.001**	**0.001**	2.458 (1.450–4.167)	** < 0.001**	**0.002**	2.333 (1.371–3.971)

Abbreviations: AC: ampullary carcinoma; OS: overall survival; RFS: recurrence-free survival; TBIL: total bilirubin; GGT: gamma-glutamyltransferase; PLR: platelet to lymphocyte ratio; NLR: neutrophil to lymphocyte ratio; AAPR: albumin to alkaline phosphatase ratio; GPR: γ-glutamate transpeptidase to platelet ratio; GLR: γ-glutamate transpeptidase to lymphocyte ratio; AGR: albumin to γ-glutamate transpeptidase ratio; CA19-9: carbohydrate antigen 19–9; IQR: Inter Quartile Range; AJCC: American Joint Committee on Cancer; NS: no significance; NA: not applicable; HR: hazard ratio; CI: confidence intervals; P<0.05 marked in bold font shows statistical significant

### Correlation between CA19-9/GGT and clinicopathological characteristics of patients

CA19-9/GGT was obtained by the patient's first laboratory examination after hospitalization, and its optimal cut-off value was obtained using the receiver operating characteristic (ROC) curve, which was approximately 0.14 [area under the curve (AUC) = 0.839, 95% CI 0.779–0.899]. The corresponding sensitivity was 87.7%, and the specificity was 68.5%. Patients were divided into two groups by the optimal cut-off value of CA19-9 / GGT: low-risk group (CA19-9/GGT ≤ 0.14, n = 69) and high-risk group (CA19-9/GGT > 0.14, n = 93) (Table 2). Compared with the low-risk group, the high-risk group patients (CA19-9/GGT > 0.14) were presented with more postoperative adjuvant therapy (*P* = 0.025), lymph node metastasis (*P* = 0.036), and nerve invasion (*P* = 0.002); lower differentiation grade (*P* = 0.001); higher levels of total bilirubin (*P* = 0.011) and CA19-9 (*P* < 0.001) and more advanced TNM stage (*P* < 0.001).

**Table 2:** Correlation between carbohydrate antigen 19–9 to gamma-glutamyltransferase ratio and clinicopathological characteristics in AC

Variables	CA19-9/GGT > 0.14 (n = 93)	CA19-9/GGT ≤ 0.14 (n = 69)	*P* value
Gender, male/female	56/37	38/31	0.512
Age, years (x ± s)	62.8 ± 8.5	60.3 ± 8.2	0.134
Preoperative jaundice reduction yes/no	24/69	14/55	0.413
Underlying diseases yes/no	43/50	26/43	0.276
Hypertension / Diabetes / Coronary heart disease / Cerebral infarction	25/19/5/8	18/11/4/5	
Hepatobiliary and Pancreatic Coexisting Diseases yes/no	35/58	18/51	0.121
Complications yes/no	51/42	37/32	0.878
Pancreatic-fistula/Gastrointestinal hemorrhage/Abdominal infection/ Gastric emptying disorder	42/10/3/8	30/6/5/5	
Postoperative adjuvant therapy yes/no	57/36	30/39	0.025
Differentiation Low/Medium/High	28/61/4	7/55/7	0.001
Lymph node invasion yes/no	36/57	16/53	0.036
Perineural invasion yes/no	44/49	16/53	0.002
Vascular invasion yes/no	10/83	7/62	0.901
AJCC 8th edition 0/ IA, B/IIA, B/IIIA, B	0/20/37/36	2/37/14/16	< 0.001
TBIL (µmol/L)>256.6 / ≤ 256.6	28/65	9/60	0.011
Albumin (g/L) > 43.55 / ≤ 43.55	9/84	7/62	0.921
Alkaline phosphatase (U/L) > 430.45 / ≤ 430.45	45/48	29/40	0.422
GGT (U/L)>86.85 / ≤ 86.85	85/8	10.5	0.983
CA19-9 (U/L)>74.97 / ≤ 74.97	73/20	22/47	< 0.001

AC: ampullary carcinoma; TBIL: total bilirubin; GGT: gamma-glutamyltransferase; CA19-9: carbohydrate antigen 19–9; AJCC: American Joint Committee on Cancer; *P* < 0.05 marked in bold font shows statistical significant

### CA19-9/GGT is a significant prognostic indicator

Receiver operating characteristic (ROC) curve was used to calculate optimal cutoff values for predictors. Cox regression model was used for survival analysis of risk factors (Table 1). In univariate analysis, gender (*P* = 0.023), tumor size (*P* = 0.023), differentiation extent (*P* = 0.023), lymph node metastasis (P = 0.004), nerve invasion (*P* = 0.001), AJCC 8th edition TNM stage (*P* < 0.001), total bilirubin (*P* < 0.001), CA19-9 (*P* < 0.001), PLR (*P* = 0.027), GPR (*P* = 0.008), GLR (*P* = 0.004), AGR (*P* = 0.003), CA19-9/GGT (*P* < 0.001) were identified as risk factors for OS (Table 1); gender (*P* = 0.027), tumor size (*P* = 0.002), differentiation extent (*P* < 0.001), lymph node metastasis (*P* < 0.001), nerve invasion (*P* = 0.004), AJCC 8th edition TNM staging (*P* < 0.001), total bilirubin (*P* < 0.001), CA19-9 (*P* < 0.001), GPR (*P* = 0.011), GLR (*P* = 0.003), AGR (*P* = 0.005), CA19-9/GGT (*P* < 0.001) affected postoperative recurrence-free time (RFS) (Table 1).

**Fig. 1:**
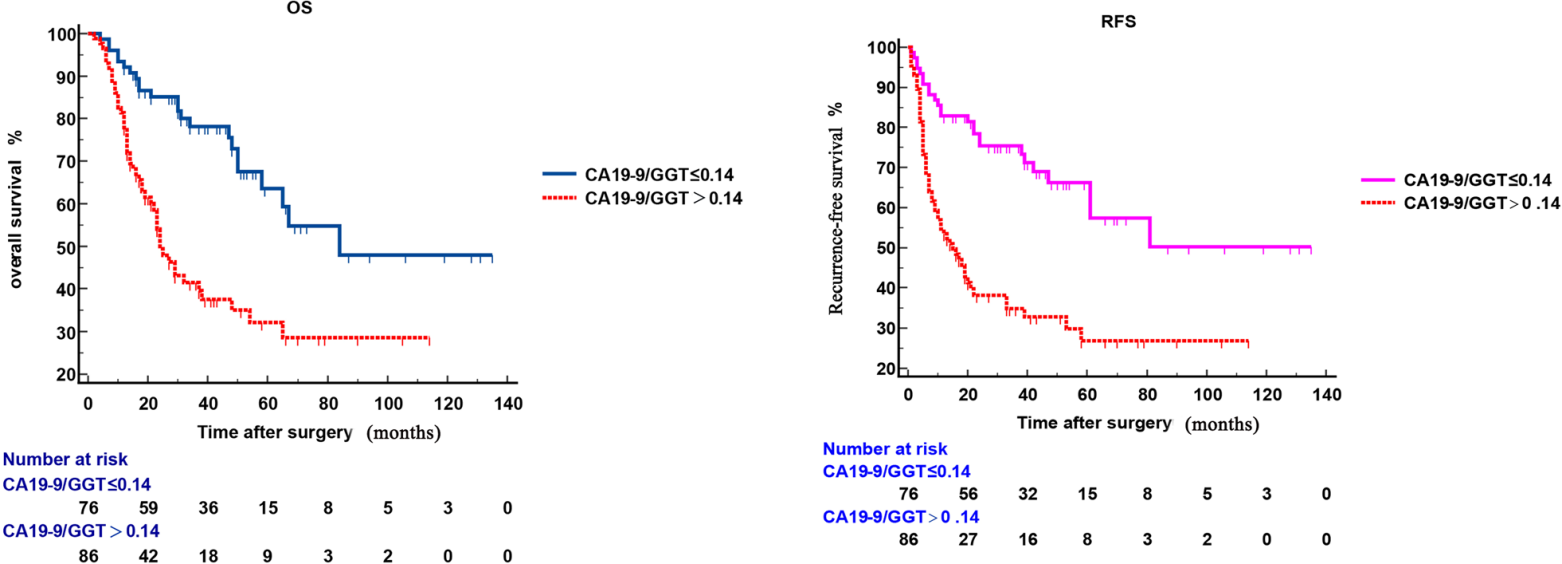
Kaplan–Meier survival curves for patients with AC stratified by CA19-9/GGT. AC patients with a preoperative CA19-9/GGT higher than 0.14 were associated with significantly poorer overall survival (**a**) and recurrence-free survival (**b**) compared with AC patients with a preoperative CA19-9/GGT lower than 0.14

In multivariate analysis, the independent risk factors affecting OS were CA19-9/GGT (*P* = 0.001, HR 2.459, 95% CI 1.450–4.167), medium/low differentiation (*P* < 0.001, HR 6.372, 95% CI 1.470–27.610), AGR (*P* < 0.001, HR 6.474, 95% CI 2.972–13.850), CA19-9 (*P* < 0.001, HR 3.401, 95% CI 1.953–5.922); the independent risk factors affecting RFS included CA19-9/GGT (P = 0.002, HR 2.333, 95% CI 1.371–3.971), medium / low differentiation (*P* < 0.001, HR 4.760, 95% CI 1.077–21.030), lymph node metastasis (P = 0.022, HR 1.762, 95% CI 1.089–2.849), CA19-9 (*P* < 0.001, HR 3.477, 95% CI 1.991–6.073), AGR (*P* < 0.001, HR 5.582, 95% CI 2.576–12.093). CA19-9/GGT > 0.14 was significantly correlated with poor prognosis of OS (*P* < 0.001) and RFS (*P* < 0.001) (Fig. 1).

### Performance comparison of CA19-9/GGT and other prognostic indicators

CA19-9/GGT, other inflammation-based scores, routine laboratory parameters and AJCC 8th staging system were used to evaluate the predictive ability by the concordance index (C-index) (Table 3). Compared with other prediction parameters, CA19-9/GGT had the highest C-index for OS and RFS prediction, which was 0.753 ( 95% CI 0.748–0.838) and 0.745 ( 95% CI 0.737–0.827), respectively. In terms of prediction accuracy, CA19-9/GGT was significantly superior to other inflammatory scores and CA19-9 in OS and RFS prediction.

**Table 3:** Performance comparison of AJCC 8th edition TNM staging, prognostic indicators and predictive models in AC patients: C index of OS and RFS

Variables	OS			RFS		
	C-index	95% CI		C-index	95%CI	
Clinicopathological characteristics						
Differentiation	0.753	0.67	0.836	0.74	0.657	0.823
Lymph node invasion	0.655	0.546	0.765	0.7	0.602	0.798
AJCC staging system						
Nomogram (AJCC 8th edition TNM staging + CA19-9/GGT)	0.787	0.685	0.712	0.795	0.696	0.713
AJCC 8th edition TNM staging	0.759	0.632	0.785	0.772	0.659	0.805
Prognostic index score						
PLR(>266.44/ ≤ 266.44)	0.682	0.557	0.807	0.664	0.542	0.786
NLR (>3.51/ ≤ 3.51)	0.587	0.468	0.708	0.59	0.479	0.702
AAPR (>0.28/ ≤ 0.28)	0.533	0.385	0.684	0.519	0.378	0.66
GPR(>4.06/ ≤ 4.06)	0.651	0.571	0.871	0.626	0.476	0.776
GLR (>507.2/ ≤ 507.2)	0.632	0.508	0.757	0.608	0.491	0.725
AGR (>0.035/ ≤ 0.035)	0.662	0.565	0.877	0.63	0.479	0.781
CA19-9/GGT (>0.14/ ≤ 0.14)	0.753	0.748	0.838	0.745	0.737	0.827
Conventional hematological indexes						
TBIL (>256.6/ ≤ 256.6 µmol/L)	0.68	0.603	0.816	0.686	0.62	0.812
Albumin (>43.55/ ≤ 43.55 g/L)	0.536	0.362	0.709	0.554	0.386	0.722
Alkaline phosphatase (>430.45/ ≤ 430.45U/L)	0.544	0.422	0.667	0.544	0.429	0.659
GGT (>86.85/ ≤ 86.85U/L)	0.526	0.306	0.747	0.552	0.348	0.755
CA19-9 (>74.97/ ≤ 74.97U/L)	0.706	0.64	0.851	0.701	0.64	0.843

AC: ampullary carcinoma; OS: overall survival; RFS: recurrence-free survival; TBIL: total bilirubin; GGT: gamma-glutamyltransferase; PLR: platelet to lymphocyte ratio; NLR: neutrophil to lymphocyte ratio; AAPR: lbumin to alkaline phosphatase ratio; GPR: γ-glutamate transpeptidase to platelet ratio; GLR: γ-glutamate transpeptidase to lymphocyte ratio; AGR: albumin to γ-glutamate transpeptidase ratio; CA19-9: carbohydrate antigen 19–9; AJCC: American Joint Committee on Cancer; TNM: Tumor, Nodes, Metastases; CI confidence intervals

### Prognostic nomograms integrating CA19-9/GGT and the AJCC 8th TNM staging systems

**Fig. 2:**
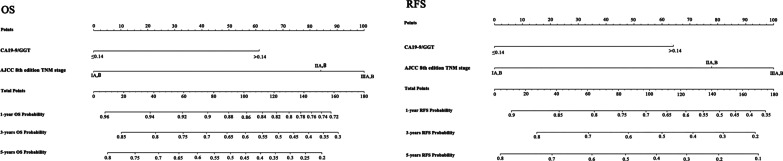
Prognostic nomogram for patients with AC. The nomogram provides a method for calculating the 1-, 3- and 5-year OS and RFS probabilities of AC based on AJCC 8th edition staging system and CA19-9/GGT. When using the nomogram, each variable axis has a patient ' s value, and draw a line up to determine the number of points for each variable value. The sum of these numbers is located on the total axis and a line is drawn down to the survival axis to determine the possibility of 1-, 3-, and 5-year OS and RFS

A new Nomogram prediction model was constructed by combining the AJCC 8th TNM staging system with CA19-9/GGT (Fig. 2). The C-index derived from the traditional AJCC 8th staging system and the Nomogram prediction model has been entered in Table 3. Compared with AJCC 8th TNM staging system (OS: C-index = 0.759, 95%CI = 0.632–0.785; RFS: C-index = 0.772, 95%CI = 0.659–0.805), the C-index of Nomogram prediction model in OS (C-index = 0.787, 95%CI = 0.685–0.712) and RFS (C-index = 0.795, 95%CI = 0.696–0.713)elevated.

**Fig. 3:**
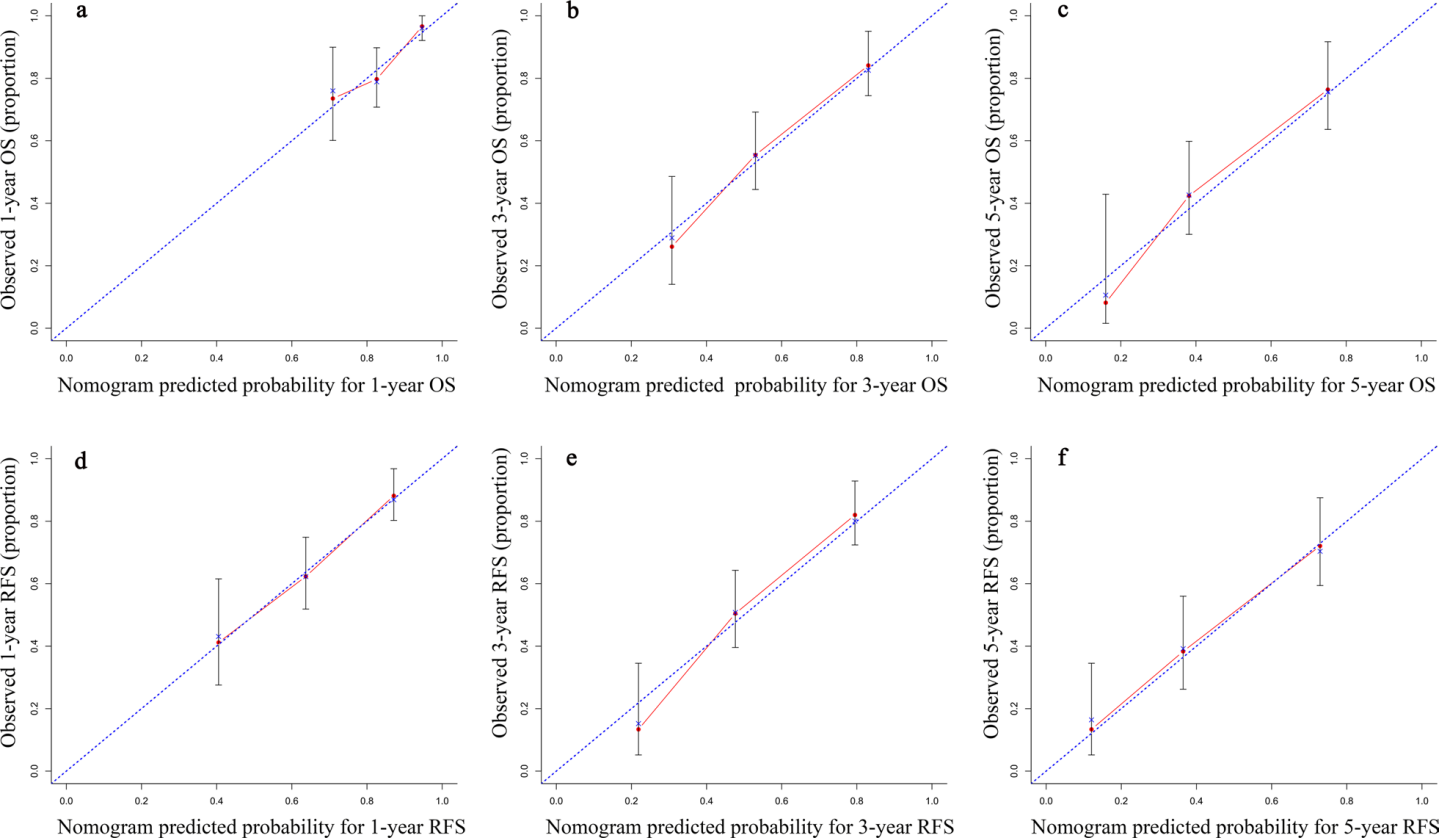
Calibration curves for OS and RFS of the prognostic nomogram. Calibration curves of the prognostic nomogram (black) for predicting OS at **a** 1 years, **b** 3 years and **c** 5 years; predicting RFS at **d** 1 years, **e** 3 years and **f** 5 years. Nomogram-predicted probability (predicted OS or RFS; x-axis) was plotted against the Kaplan–Meier estimate (observed OS or RFS; y-axis). Dashed line indicates the reference line, indicating where an ideal would lie

**Fig. 4:**
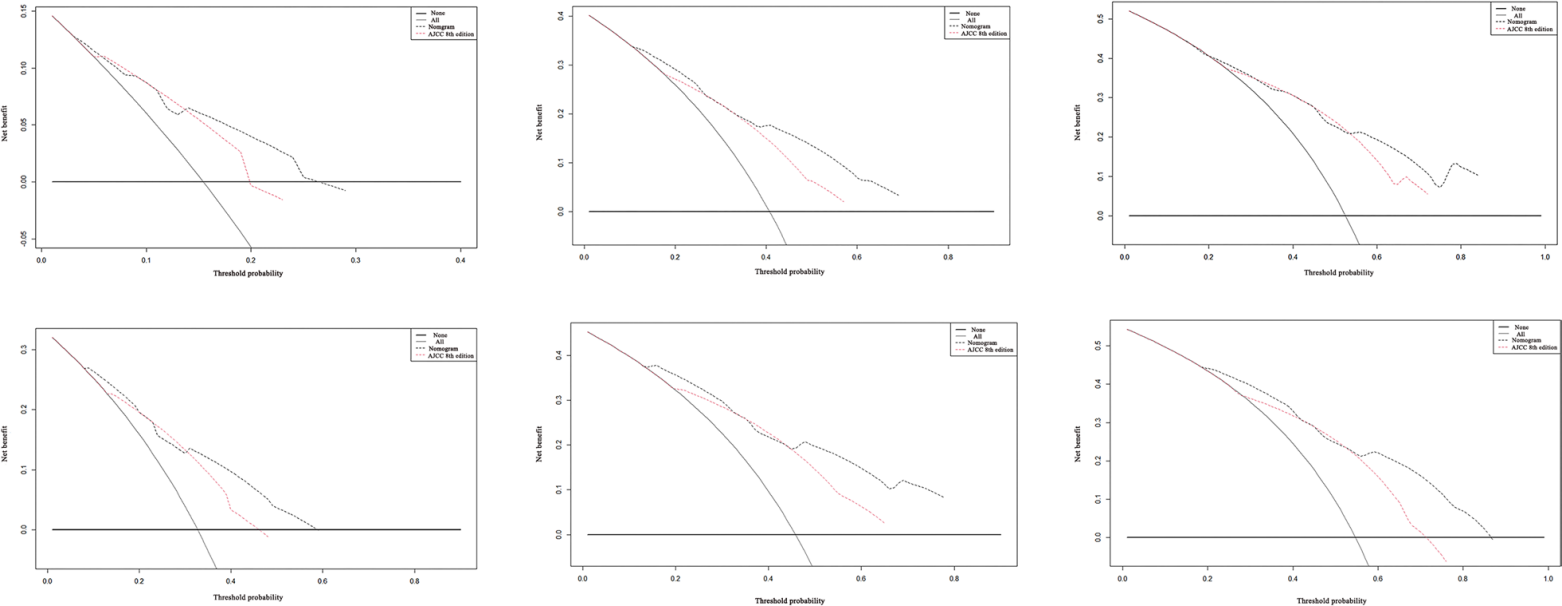
Decision curve analyses for OS and RFS of the prognostic nomogram and the simple AJCC 8th edition staging system. Dashed lines indicate the net benefit of the predictive models across a range of threshold probabilities (black: nomogram; pink: AJCC 8th edition TNM stage). The horizontal solid black line represents the assumptions that no patient will experience the event, and the solid grey line represents the assumption that all patients will experience the event. On decision curve analysis, the nomogram showed superior net benefit compared with AJCC 8th edition staging system across a wider range of threshold probabilities

The calibration curve intuitively reflected that the 1-year, 3-year, and 5-year OS and RFS of the actual observations were in good consistency with the 1-year, 3-year, 5-year OS and RFS calculated by the Nomogram prediction model (Fig. 3). Decision curve analysis (DCA), as an assessment method reflecting the clinical net benefit of the predictive model, intuitively confirms that the Nomogram predictive model involved a wider range of threshold probabilities than the traditional AJCC 8th staging system, yielded better net benefit, thereby provided higher prediction accuracy (Fig. 4, Table 4).

**Table 4:** Net benefit range of Nomogram and AJCC 8th edition TNM staging system

Variables	OS			RFS		
	Nomogram	AJCC	Nomogram > AJCC	Nomogram	AJCC	Nomogram > AJCC
1 (year)	0.01–0.26	0.01–0.19	0.14–0.19	0.01–0.58	0.01–0.46	0.31–0.58
3 (year)	0.01–0.69	0.01–0.57	0.12–0.69	0.01–0.78	0.01–0.65	0.15–0.78
5 (year)	0.2–0.84	0.25–0.72	0.55–0.84	0.01–0.86	0.01–0.71	0.18–0.43, 0.53–0.86

AJCC: American Joint Committee on Cancer; TNM: Tumor-Node-Metastasis; OS: overall survival; RFS: recurrence-free survival

## Discussion

This study hypothesized and established a new and more predictive parameter (CA19-9/GGT) as a prognostic indicator. A series of scores based on inflammatory factors have been used as convenient and feasible prognostic indicators for various malignant tumors in the past decade [22–24]. However, due to the rareness of ampullary carcinoma and the possibility of being affected by coexisting diseases, such as obstructive jaundice and cholangitis, the predictive value of these inflammation-based scores is less reliable [1, 12–14, 19]. This study is the first attempt to use this prognostic indicator of CA19-9 to GGT ratio to predict the prognosis and survival of patients with ampullary carcinoma after radical resection.

Serum carbohydrate antigen (CA) 19–9, as one of the most common tumor markers in clinic, is significantly correlated with the diagnosis, postoperative recurrence and metastasis, and long-term prognosis of malignant tumors such as distal cholangiocarcinoma, gallbladder cancer, pancreatic cancer, and ampullary cancer [25, 26]. In this study, CA19-9 (*P* < 0.001, HR 3.477, 95% CI 1.991–6.073) was also confirmed as an independent risk factor for postoperative long-term survival in patients with ampullary carcinoma through univariate and multivariate analysis However, when biliary tract lesions occur, biliary epithelial cells are damaged and permeability increases, and then CA19-9 will be abnormally elevated [7, 8]. In addition, the treatment of reducing jaundice can also lead to a significant decline in CA19-9 [7, 8]. Therefore, the ability of CA19-9 to diagnose and predict the prognosis of ampullary carcinoma is greatly weakened, and its specificity is reduced, thereby reducing the predictive value of long-term survival of patients [8, 27].

Previous studies have reported the correlation between serum γ-glutamyltransferase (GGT) level and cancer risk. For the relationship between GGT and cancer, a potential mechanism has been hypothesized [28–30]: As a key enzyme in glutathione (GSH) metabolism, GGT is the main antioxidant of cells, which catalyzes the degradation of extracellular GSH and plays a key role in neutralizing reactive oxygen species and free radicals; In addition, GGT is an important part of the cellular defense system, GGT and GSH are used to resist oxidative stress. Elevated GGT levels in tumor cells can generate reactive oxygen species (ROS), which may promote tumor progression. However, through the univariate analysis of this study, it was found that GGT was not an independent risk factor affecting the long-term survival of patients with ampullary carcinoma (*P* > 0.05). In this regard, a large number of studies are needed in the future to verify and analyze the correlation between GGT and prognosis and survival.

The CA19-9/GGT ratio can not only calibrate the serum CA19-9 concentration by GGT to reduce the influence of biliary obstruction or other factors on the serum CA19-9 level, and then evaluate the long-term survival ability of patients with radical resection of ampullary carcinoma, but also is a new, easily accessible and more potential prognostic indicator. In this study, univariate and multivariate analysis confirmed that CA19-9/GGT, medium / low differentiation, AGR and CA19-9 remained as independent risk factors affecting OS; CA19-9/GGT, medium / low differentiation, lymph node metastasis, CA19-9, and AGR remained as independent risk factors affecting RFS. All patients were divided into two subgroups according to the optimal cut-off value (0.14) of CA19-9/GGT. Compared with the subgroup ≤ 0.14, the patients in the subgroup with CA19-9/GGT > 0.14 received more adjuvant therapy, more lymph nodes Invasion, more nerve invasion, higher levels of total bilirubin and CA19-9, lower degree of differentiation, and later TNM stage, which proved that the higher the CA19-9 / GGT ratio, the worse the postoperative long-term survival. Therefore, CA19-9/GGT was considered as an independent risk factor for OS and RFS in this study.

Subsequently, in order to further verify the reliability and accuracy of CA19-9/GGT, we counted the C-index in R. According to the statistical results, we concluded that the predictive ability of CA19-9/GGT (OS: C-index = 0.753, RFS: C-index = 0.745) was significantly higher than other inflammation-based scores, CA19-9 and conventional blood indexes. In addition, the Nomogram was established by incorporating CA19-9/GGT into the AJCC 8th TNM staging system, and the concordance index of Nomogram as a prediction model was compared with that of the traditional AJCC staging system (OS: C-index = 0.759, RFS: C-index = 0.772). The results show that the Nomogram prediction model (OS: C-index = 0.787, RFS: C-index = 0.795) provides higher prediction accuracy, improves the prediction performance of the traditional AJCC staging system, and also shows the predictive value of CA19-9/GGT for the long-term survival of AC patients after radical resection. The reliability and accuracy of the Nomogram prediction model were further verified by calibration curves and DCA. Therefore, it is logical to regard CA19-9/GGT as a newer, more potential and more predictive prognostic indicator for the long-term survival of patients with AC compared with previous inflammation-based scores. At the same time, the research results support the integration of CA19-9/GGT into the traditional AJCC staging system to improve its prediction performance and prediction accuracy.

This study also has its own limitations. First of all, this study is a retrospective study. The input samples are regional, and the risk factors in this region are different from other regions [31]. In addition, this database only includes patients with ampullary carcinoma who underwent radical surgery. Other patients who did not meet this condition were not verified, and the sample size was small, without external verification.

In conclusion, this study confirms that CA19-9/GGT is a new, readily available, more reliable predictive parameter with potential predictive value. In addition, the Nomogram prediction model established by incorporating CA19-9/GGT into the AJCC 8th TNM staging system showed higher prediction accuracy compared with the traditional AJCC staging system, improved the prediction performance of the traditional AJCC staging system, and also it further reflects that CA19-9/GGT has certain predictive value. At the same time, it provides additional evidence for surgeons to make better clinical decisions before surgery, and theoretically helps predict patient prognosis and survival.

## Data Availability

The datasets used and/or analyzed during the current study are available from the corresponding author on reasonable request.
